# Hematotoxicity Induced by Copper Oxide Nanoparticles and the Attenuating Role of Giloy In Vivo

**DOI:** 10.7759/cureus.46577

**Published:** 2023-10-06

**Authors:** Ozdan Akram Ghareeb

**Affiliations:** 1 Department of Community Health, Northern Technical University, Kirkuk, IRQ

**Keywords:** alternative medicine, health, experiment, oxide nanoparticles, hematotoxicity

## Abstract

Background

In line with the growing industrial applications of copper oxide nanoparticles (CuONPs) in various fields, concerns about their potentially harmful consequences on the environment, human, and animal health are increasing. Giloy is considered an alternative medicine to treat various ailments. Giloy's potential in helping manage diabetes, alleviating arthritis and joint pain, and addressing skin disorders such as eczema and acne underscores its multifaceted role in traditional medicine. Moreover, it is deemed beneficial for reducing stress and anxiety levels, promoting liver health, and potentially impacting heart health by regulating cholesterol levels. Emerging research also explores its potential in cancer prevention. This study aimed to evaluate the hematotoxicity of CuONPs and the alleviating effect of giloy in adult rats.

Materials and methods

In this experiment, 28 laboratory rats were used, set to four groups (7/group), as follows: control group without any dose; CuONPs group administered copper oxide nanoparticles at 300 mg/kg/day; CuONPs + giloy group dosed with CuONPs at 300 mg/kg/day plus giloy at 100 mg/kg/day; giloy group treated only with giloy at 100 mg/kg/day. All treatments were given by gastric gavage and continued for 28 uninterrupted days.

Results

Dosing animals with CuONPs led to significant adverse changes in the examined blood profile. In contrast, when the animals were coadministered with giloy, restoring the disturbed blood levels was observed.

Conclusion

Copper oxide nanoparticles at a high dose had notable hematotoxicity in laboratory rats and, supplemented with giloy, could reduce this hematological toxicity.

## Introduction

Nanomedicine has sparked a major revolution in the current era in many branches of industries like biomedicine for its great role in improving the accuracy of diagnosis and facilitating the treatment of many diseases, especially intractable ones [1-3]. As nanoparticles are smaller than cellular organelles (<100 nm), they can easily penetrate body cells and make them suitable for various applications in medical fields [4]. Copper oxide nanoparticles (CuONPs) are widely applied in various modern technological, medical, and cosmetic industries due to their excellent optical and electrical properties along with antimicrobial and antioxidant abilities [5,6]. Thus, the release of these particles into the environment is relatively high, and this raises concern about its potential harmful effects on human health [7]. This is supported by the presence of several documented scientific experiments that revealed the toxic effects of metal oxide nanoparticles in laboratory animals [8-10].

In general, exposure of the body to nanoparticles can occur in several ways, reaching the blood and then spreading to other vital targets [11]. It should be noted that the administration of nanoparticles in toxic doses causes changes in blood parameters indicative of various blood disorders [12]. Therefore, the assessment of blood indices can be adopted as a key diagnosis of systemically induced detrimental impacts on the body [13]. Many people have resorted to medicinal herbs from ancient times until today, to treat health problems due to their cheapness, availability, and relatively few side effects [14]. Giloy, with the generic name Tinospora cordifolia, is a climbing shrub with flowers belonging to the Menispermaceae family. It has various medicinal advantages as an anti-inflammatory, antioxidant, antiviral, and immunomodulator [15]. Likely, it consists of various bioactive chemical compounds beneficial to body tissues including alkaloids, diterpenoids, glycosides, sitosterols, and phenolic acids [16]. This study was designed to estimate the adverse effects of CuONPs on some blood Indices and to investigate the possibility of improving the role of giloy.

## Materials and methods

Copper oxide nanoparticle (CuONP) dispersion has particle sizes ranging from 25 to 55 nm (US Research Nanomaterials, Inc., Houston), with specifications reported in Table 1.

**Table 1:** Main specifications of CuONP dispersion

Product	Copper Oxide Nanoparticles
Molecular formula	CUO
Form	Dispersion
Solvent	Water
Concentration	20 wt.%
Purity	99.95+%
PH	7-9
Color	Black
Morphology	Nearly spherical

As for the Giloy Ras Juice's herbal composition, it was sourced from a company named Herbal Canada (Uttar Pradesh, India). It consists of 100% natural giloy, known for its immune-boosting and blood-purifying properties.

Twenty-eight male albino rats, weighing 195-235 g and aged four to five months, obtained from the animal houses of Iraqi universities, were used in this study. Animals were housed in custom plastic cages with free access to water and a standard diet, with an emphasis on keeping them in an ideal laboratory environment in terms of temperature, light, humidity, and ventilation. The study began after a week of acclimating the rats to laboratory conditions. They were divided into four groups (7/group), and they were dosed as follows: control group without any treatment; CuONP group administered copper oxide nanoparticles at 300 mg/kg according to previous studies [17,18]; CuONPs+ Giloy group was treated with CuONPs plus giloy extract at 100 mg/kg [19,20]; and giloy group received giloy (100 mg/kg). All treatments were done using gastric gavage and lasted for 28 uninterrupted days.

Ethics statement

This experiment was conducted while following the guidelines of scientific and ethical research at Iraqi universities, based on procedures used by the US National Institutes of Health, which were officially certified in 1978 for research using laboratory animals with institutional review board number IEC/NTU/51.

Blood indices

All the rats in the study underwent anesthesia and were euthanized one day after receiving the final dose of treatment. Subsequently, a cardiac ventricular puncture was performed on each rat to collect blood samples using sterile equipment, which were then placed in EDTA tubes for the purpose of examining various hematological parameters. To conduct a comprehensive analysis of these parameters, a complete blood count test was conducted using an automated hematology analyzer. This test encompassed the assessment of several key components, including the count of red blood cells (RBC), levels of hemoglobin (Hb), hematocrit (HCT), mean corpuscular volume (MCV), platelets (PLT), and the count of white blood cells (WBC).

Statistical analysis

All results were analyzed by Statistical Product and Service Solutions (SPSS) (version 26; IBM SPSS Statistics for Windows, Armonk, NY) software and data expressed as means ± standard deviation. Comparisons between experiment groups were done by applying an ANOVA test, followed by a Duncan Hoc with a significant dependence at P<0.05.

## Results

**Figure 1:**
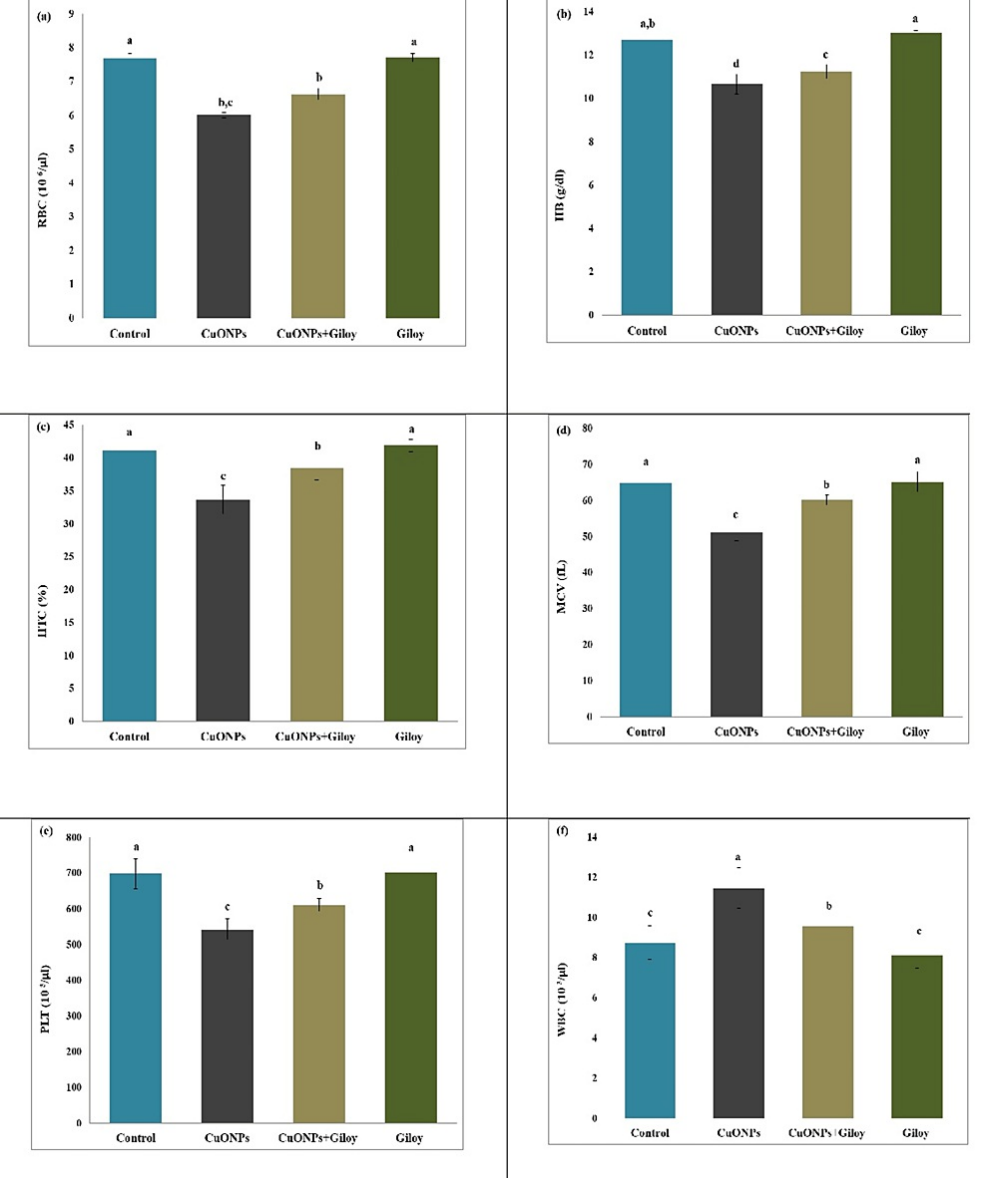
Effects of CuONPs and giloy on blood parameters: a) RBC, b) HB, c) HTC, d) MCV, e) PLT, and f) WBC in studied rats. All results are displayed as mean ± SD. Super letters (a-d) express remarkable variation between experimental groups

The results of this experiment showed that receiving CuONPs at 300 mg/kg for 28 days caused remarkable lowering (P<0.05) in the RBCs, HB, HCT, MCV, and PLTs with an increase in WBC counts compared with the control group. In contrast, the CuONPs plus giloy-treated groups showed a clear improvement in terms of all hematological indices when compared to the CuONPs group. Otherwise, no important variations were observed in hematologic indices between the control and giloy groups (Figure 1, Table 2).

**Table 2:** Comparative analysis of blood parameters in different experimental groups ^a^, ^b^, and ^c^ in a cell indicate that a group is significantly different from more than one other group RBC (106/µl): red blood cells per microliter of blood; HB (g/dl): hemoglobin in grams per deciliter of blood; HCT (%): hematocrit; MCV (fL): mean corpuscular volume in femtoliters; PLT(103/µl): platelets per microliter of blood; WBC (103/µl): white blood cells per microliter of blood

Parameters	Experiment Groups			
	Control	CuONPs	CuONPs+ Giloy	Giloy
RBC (10^6^/µl)	7.64±0.13^a^	6.12±0.11^b,c^	6.77±0.20^b^	7.83±0.16^a^
HB (g/dl)	12.71±0.21^a,b^	10.67±0.45^d^	11.24±0.32^c^	13.01±0.12^a^
HCT (%)	41.11±1.32^a^	33.65±2.15^c^	38.47±1.86^b^	41.86±0.94^a^
MCV(fL)	64.81±3.15^a^	51.25±2.35^c^	60.19±1.34^b^	65.13±2.80^a^
PLT(10^3^/µl)	704.24±38.76^a^	536.76±26.45^c^	612.57±22.14^b^	710.13±31.54^a^
WBC(10^3^/µl)	8.74±0.83^c^	11.46±1.01^a^	9.58±0.42^b^	9.57±0.65^c^

## Discussion

Generally, the blood is affected by the clinical pathological state of the body tissues, and the entry of foreign chemicals into the bloodstream and their interaction with the components of the blood cause disturbances of the functional characteristics of the blood parameters. Therefore, the evaluation of the blood profile is one of the main diagnostic indicators for the deleterious effects of toxic foreign substances through blood disorders in human and animal bodies alike [21-23]. In this study, intoxicated animals with copper oxide nanoparticles showed a clear disruption in blood indices when compared to control group. Our results are in line with previous studies confirming that treating laboratory animals with nanoparticles in toxic doses caused blood disorders [24-28]. Lee et al. demonstrated that repeated (28 days) exposure of rats to Cu nanoparticles (25 nm) at 200 mg/kg led to red blood cell destruction, which was characterized by a decrease in RBC count, HB, HCT, and body MCV, which indicated that chronic copper intoxication caused hemolytic anemia with various hematological changes that were consistent with the results of this study [29]. Decreased levels of RBCs and hemoglobin concentration are usually associated with the development of anemia. This can be the result of blood loss, hemolysis, or insufficient production of red blood cells and hemoglobin [30,31]. It is known that hematocrit expresses the viscosity of the blood, and its low level is an indication of anemia, and this may be the result of either lowering erythrocyte count or decreasing hemoglobin concentration in each red blood cell or both [32]. Mean corpuscular volume MCV is a measure of average erythrocyte volume to check for signs of medical conditions such as anemia. The reduction of MCV may be because of decreased erythrocyte formation [33]. Thrombocytopenia means that the level of platelets in blood circulation is lower than normal levels either because of decreased production by the bone marrow or increased destruction of platelets [34]. As for leukocytes, they are vulnerable to the inflammatory response, especially at the site of the presence of foreign particles to defend and restore homeostasis [35]. Changes in blood parameters in rats poisoned with CuONPs indicated the harmful effect of CuONPs on the hematopoietic system as well as stimulating immune system response. It is worth noting that the main mechanism of CuONP toxicity in body cells is the activation of reactive oxygen species (ROS) generation and stimulation of the redox system, thus inducing oxidative stress. Excessive ROS leads to inflammatory responses along with damage to cell membranes, DNA, and proteins [36,37]. Anemia diseases are closely related to oxidative damage, and antioxidant systems play a more important role in maintaining red blood cell homeostasis against oxidative insult than normal cells. As antioxidative enzymes counteract oxidative damage in erythrocyte cells, and, in turn, their absence limits the formation of erythropoiesis and the lifespan of erythrocytes, this results in the development of anemia [38].

In this experiment, the beneficial effects of giloy on induced hematotoxicity by CuONPs were observed in CuONPs + giloy rats. Our findings are in agreement with observations of several previous experimental studies in vivo that demonstrated the protective action of giloy against toxins and reducing the toxicity of various agents in body organs with their main components. Thus, it can be considered as a protective herb against many harmful chemicals [39-41]. The medicinal properties offered by giloy greatly supported its traditional medical uses, most notably for their antioxidant and anti-inflammatory properties. Based on documented reports, it is believed that the chemical components in giloy may activate Nrf2, leading to an overexpression of various antioxidant enzymes and thus triggering an adaptive response to oxidative stress [42]. In a previous experimental study by Kumar et al. (2020), they concluded that the ethanolic extract of the giloy plant (400 mg/kg) had a crucial defensive role in arsenic-induced toxicity in rats at hematological and biochemical levels [43]. Ghatpande et al. (2019) found that T. cordifolia had a protective ability against inflammatory anemia as well as maintained iron turnover by reducing inflammatory cytokines and hepcidin expression in experimental rats. The treated animals with T. cordifolia showed significantly higher Hb and RBC levels than the inflammation group [44]. Alrumaihi and colleagues (2019) suggested that treatment with T. cordifolia (100 mg/kg) regenerates a weak immune system and eradicates systemic candidiasis in rats through its ability to restore leukocytes [45].

The study has some limitations such as the study lasting for 28 days, providing insights into short-term effects. Long-term effects and potential accumulative impacts over extended periods were not addressed. The study concentrated on copper oxide nanoparticles. Different types of nanoparticles with distinct properties may induce diverse biological responses. Therefore, the findings might not be generalizable to other nanoparticle types. The study's conclusions are drawn from rat experiments. Direct human data are essential to validate the potential health implications of copper oxide nanoparticles and giloy supplementation in humans. The study did not consider the environmental factors that could influence nanoparticle toxicity. Nanoparticles' behavior and effects in real-world environmental conditions might differ from laboratory settings.

## Conclusions

In summary, this study provides valuable insights into the hematotoxicity of CuONPs in adult rats and highlights the potential of giloy as a complementary treatment to ameliorate these toxic effects. However, coadministration with giloy clearly mitigated this toxicity. Therefore, it can be recommended as a promising protective agent against nanoparticle-induced haematotoxicity and also against other toxins and chemicals. There is a need for more experiments dealing with other tissues of the body.
